# Patterns and outcomes of patients with abdominal trauma on operative management from northern Tanzania: a prospective single centre observational study

**DOI:** 10.1186/s12893-019-0530-8

**Published:** 2019-06-26

**Authors:** Shilanaiman Hilary Ntundu, Ayesiga M. Herman, Alfred Kishe, Heri Babu, Ola F. Jahanpour, David Msuya, Samuel G. Chugulu, Kondo Chilonga

**Affiliations:** 1 Department of General surgery, Kilimanjaro Christian Medical Centre, P.O. Box 3010, Moshi, Tanzania; 2 Kilimanjaro Christian Medical University College, P.O Box 2240, Moshi, Tanzania; 3 School of Public Health, Department of Epidemiology and Biostatistics, Catholic University of Health and Allied Sciences, Mwanza, Tanzania

**Keywords:** Abdominal trauma, Patterns, Outcomes

## Abstract

**Background:**

The abdomen is one of the most commonly injured regions in trauma patients. Abdominal injury surgeries are common in Tanzania and in many parts of the world.

This study aimed to determine the relationships among the causes, characteristics, patterns and outcomes of abdominal injury patients undergoing operations at Kilimanjaro Christian Medical Centre.

**Methods:**

A prospective observational study was performed over a period of 1 year from August 2016 to August 2017. A case was defined as a trauma patient with abdominal injuries admitted to the general surgery department and undergoing an operation. We assessed injury types, patterns, aetiologies and outcomes within 30 days. The outcomes were post-operative complications and mortality. Multivariate logistic regression was used to explore the association between factors associated with morbidity and mortality.

**Results:**

Out of 136 patients, 115 (84.6%) were male, with a male-to-female ratio of 5.5:1. The most affected patients were in the age range of 21–40 years old, which accounted for 67 patients (49.3%), with a median age (IQR) of 31.5 (21.3–44.8) years. A majority (99 patients; 72.8%) had blunt abdominal injury, with a blunt-to-penetrating ratio of 2.7:1. The most common cause of injury was road traffic accidents (RTAs; 73 patients; 53.7%). Commonly injured organs in blunt and penetrating injuries were, respectively, the spleen (33 patients; 91.7%) and small bowel (12 patients; 46.1%). Most patients (89; 65.4%) had associated extra-abdominal injuries. Post-operative complications were observed in 57 patients (41.9%), and the mortality rate was 18 patients (13.2%). In the univariate analysis, the following were significantly associated with mortality: associated extra-abdominal injury (odds ratio (OR): 4.9; *P*-value< 0.039); head injury (OR: 4.4; *P*-value < 0.005); pelvic injury (OR: 3.9; *P*-value< 0.043); length of hospital stay (LOS) ≥ 7 days (OR: 4.2; *P*-value < 0.022); severe injury on the New Injury Severity Score (NISS) (OR: 21.7; *P*-value < 0.003); time > 6 h from injury to admission (OR: 4.4; *P*-value < 0.025); systolic BP < 90 (OR: 3.5; *P*-value < 0.015); and anaemia (OR: 4.7; *P*-value< 0.006). After adjustment, the following significantly predicted mortality: severe injury on the NISS (17 patients; 25.8%; adjusted odds ratio (aOR): 15.5, 95% CI: 1.5–160, *P*-value < 0.02) and time > 6 h from injury to admission (15 patients; 19.2%; aOR: 4.3, 95% CI: 1.0–18.9, *P*-value < 0.05).

**Conclusion:**

Blunt abdominal injury was common and mostly associated with RTAs. Associated extra-abdominal injury, injury to the head or pelvis, LOS ≥ 7 days, systolic BP < 90 and anaemia were associated with mortality. Severe injury on the NISS and time > 6 h from injury to admission significantly predicted mortality.

## Background

Trauma is the leading cause of morbidity and mortality in younger populations worldwide [1, 2]. It is estimated that by the year 2020, 8.1 million people will die yearly as a result of injuries, and road traffic accidents (RTA) will be the third-most common cause of disabilities globally and the second-most common cause in developing countries [3].

Trauma accounts for major losses of the workforce due to the associated morbidity and mortality. It was estimated that approximately 671 billion dollars were spent on trauma victims in the United States of America in 2013, and the costs associated with fatal injuries are 214 billion dollars [4].

For the past 30 years in Africa, there has been an increase in RTA-related injuries and deaths [5]. The African region had a higher rate of mortality from road traffic injuries worldwide, at 26.6 per 100,000 population in 2013 [6]. In 2013, over 85% of all deaths and 90% of disability-adjusted life years (DALYs) lost from road traffic injuries occurred in low- and middle-income countries (LMICs), which have only 47% of the world’s registered vehicles [6, 7]. In South Africa, approximately 50,000 mortalities related to injuries were reported, with the majority relating to violence and RTAs [8].

Trauma management in the developing world is faced with many challenges. The observed rising trend of injuries, especially in Africa, has been linked to poor infrastructure and urbanization [9]. Inadequate and underdeveloped emergency systems to care for victims further amplify the magnitude of the problem because there are no well-established response teams in most places. In this scenario, an abdominal trauma victim’s outcome becomes catastrophic [9].

In developed countries, trauma victims have better outcomes because of the costly trauma care centres with multidisciplinary teams caring for the victims. The implementation of policies to prevent or reduce the occurrence of trauma has recently come into play [10]. The WHO has developed trauma care guidelines for LMICs, where 90% of the incidence and an even higher number of mortalities for trauma victims are reported [10, 11]. The guidelines are for essential trauma care, pre-hospital trauma care, and trauma quality improvement programmes. Studies from South Africa and Botswana have assessed the implementation of the guidelines and have developed a model recommended for LMICs. This model uses primary health facilities (Level IV), district hospitals (Level III), regional referral hospitals (Level II), and a trauma centre in a consultant hospital (Level I) with different specialities to care for trauma victims. In this system, the referral system can be bypassed, and trauma victims are taken to a centre well equipped for trauma care after resuscitation and the prevention of delays in appropriate care [12, 13].

It is estimated that approximately one-third of all trauma patients have abdominal injuries. These injuries require careful triaging for appropriate intervention because approximately 25% of such injuries require surgery [2].

The most common abdominal injuries are blunt and penetrating, and commonly injured organs are the spleen, bowels, stomach, and liver, with the least-frequently injured organs being the diaphragm and kidneys [14].

Despite injuries being one of the leading causes of morbidity and mortality in the developing world, little attention has been given to interventions designed to halt the occurrence of these injuries and the management of the victims. This study was conducted to highlight the trend of abdominal trauma and to determine the relationship between causes and injury patterns and outcomes for patients requiring surgical intervention at Kilimanjaro Christian Medical Centre (KCMC).

## Methods

### Study design and study area

This was a one-year prospective observational study on the operative management of patients with abdominal trauma presenting at the accident and emergency (A&E) department of KCMC from August 2016 to August 2017. KCMC is a tertiary teaching hospital situated in the north-eastern zone of Tanzania in the Kilimanjaro region.

### Study participant’s characteristics

We included all patients undergoing operations with a diagnosis of abdominal trauma presenting at the A&E department, general surgery, orthopaedic wards, and surgical intensive care units (ICUs). Patients who were not able to provide a proper history about the trauma, who had no accompanying relative to provide consent, who were operated on at another health facility and later referred to KCMC, and who were on non-operative management were excluded from the study.

The recruitment of patients to participate in the study was performed at the A&E department. Patients were screened for the inclusion criteria and enrolled in the study after informed consent was obtained.

Patients were resuscitated at the A&E department according to the ATLS and were then taken to the general surgery ward, to the ICU or directly to the operating theatre for the continuation of care and treatment.

The severity of injuries was determined using the New Injury Severity Score (NISS). Mild injury consisted of NISS < 16, moderate injury NISS 16–24 and severe injury NISS ≥25 [15]. The systolic BP was recorded at the A&E department with two categories: ≥ 90 mmHg and < 90 mmHg. Patients with head injuries were categorized according to the Glasgow Coma Scale (GCS). The categories were mild (13–15), moderate (9–12), and severe (3–8). Other investigations, haematocrit levels, blood grouping and cross match, X-rays, abdominal ultrasound, and CT scans were performed on admission.

All patients were operated and followed-up for 30 days or until death. Patients were visited on the 1st day, 3rd day, 7th day and 30th day for evaluation.

A pre-tested questionnaire was used to collect the information. We included in the questionnaire the following: socio-demographic data (age, sex, level of education and health insurance status); injury mechanism; pre-hospital care; interval time from injury to admission; systolic blood pressure (BP); pulse rate (PR); type, aetiology and pattern of injury; trauma score; associated extra-abdominal injury; treatment executed; and complications on arrival. The outcomes were post-operative complications, length of stay (LOS) and mortality.

### Reporting

Results are reported according to Strengthening the Reporting of Observational Studies in Epidemiology (STROBE) guidelines [16].

### Data analysis

We used SPSS software version 25.0 (IBM SPSS statistics for Windows NY; IBM Corp) to perform statistical data analysis. For categorical variables, data were summarized in proportions and frequency tables. For continuous variables, we used ranges, medians and inter-quartile ranges (IQRs) to summarize the data. We computed *P*-values for categorical variables using chi-square (X^2^) and Fisher’s exact tests in accordance with the size of the dataset. We used an independent Student’s t-test for continuous variables. We determined the variables associated with the outcome using logistic regression. A *P*-value of < 0.05 was considered to be significant.

## Results

### Patients and clinical characteristics

**Fig. 1:**
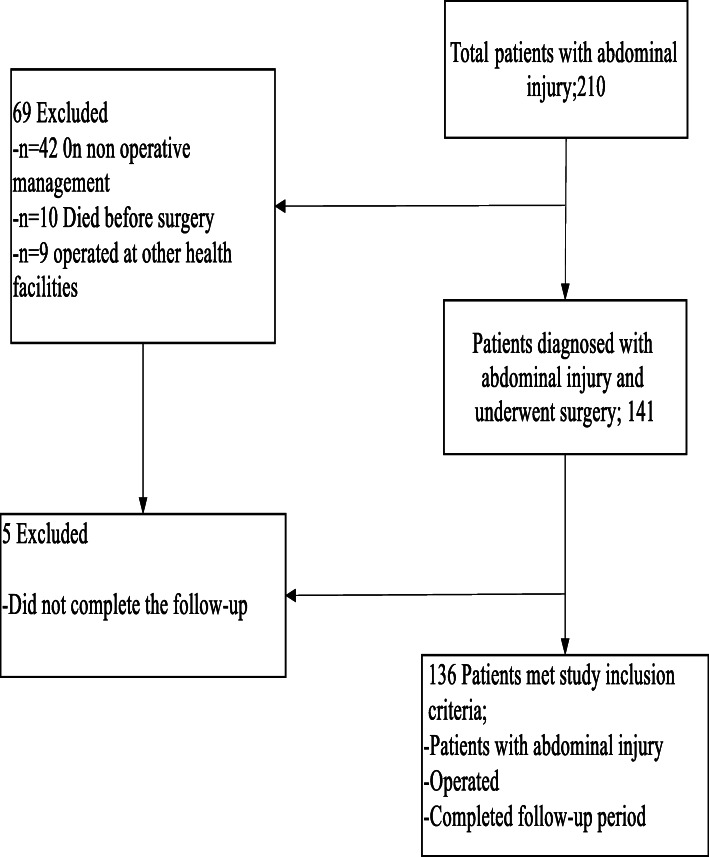
Flow diagram of patient inclusion and exclusion

During the study period, a total of 2112 trauma patients were admitted. Among these patients, 210 had abdominal injuries, 42 on non-operative management, 10 died before surgery, 9 operated at another health facility and 141 underwent operations; these patients comprised the study population. Five patients did not complete the follow-up and were excluded from the final analysis (Fig. 1).

The participants were between 2 and 95 years of age and had a median (IQR) age of 31.5 (21.3–44.8) years. The peak age incidence of the majority ranged between 21 to 40 years, accounting for 67 cases (49.3%), and 38 patients (27.9%) were aged above 40 years. Most of the study participants were male 115(84.6%) patients, with a male-to-female ratio of 5.5:1. Approximately two-thirds (66.2%) had a primary level of education, and a majority of 123 (90.4%) had no health insurance. Three-quarters of patients used an ambulance as a mode of transportation to the hospital, and 26 (19.1%) used public transportation. More than half of the participants 73(53.7%) had RTAs as a cause of injury, and 29 (21.3%) fell from a height. A total of 89 patients (65.4%) had associated extra-abdominal injuries (Table 1).

**Table 1:** Patients’ short-term outcomes

Patients Outcomes	n	%
SSI	20	28
Anaemia	15	21
Systolic blood pressure		
<90 (shock)	41	30.1
Burst abdomen	3	5.3
Intestinal obstruction	3	5.3
Paralytic ileus	3	5.3
Comatose	1	1.8
UTI	3	5.3
Intra-abdominal abscesses	2	3.5
Convulsions	2	3.5
Wound re-bleed	2	3.5
Pneumonia	2	3.5

### Injury background

The chest was the most commonly injured extra-abdominal region 46(33.8%) patients; followed by the head 42 (30.8%) patients and the extremities 37 (27.2%) patients. (Table 3).

In this study, 66 (48.5%) patients had severe injuries on the NISS scale. A total of 27(19.8%) patients had moderate to severe head injuries, and 5(3.7%) patients had severe head injuries on the GCS. On admission, 41(30.1%) patients had a systolic blood pressure < 90 (Table 1).

### Injury pattern among patients with abdominal injuries

**Fig. 2:**
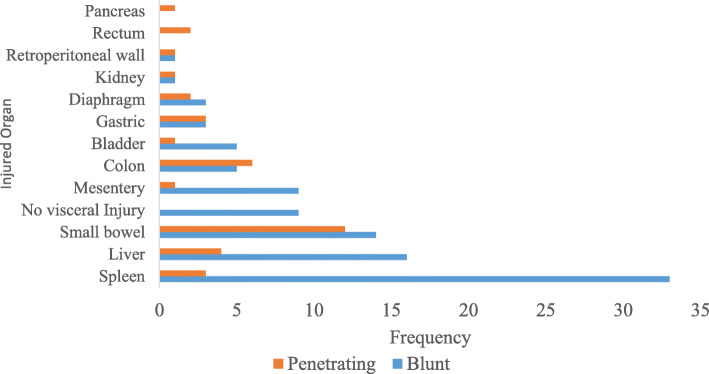
Patient injury pattern according to visceral organ injury

All 136 patients underwent operation for either blunt or penetrating abdominal injury. The spleen was the most commonly injured organ 33 (91.7%) patients in blunt injury, followed by the liver 16 (80%) patients and the small bowel 14(56.0%) patients. The retroperitoneal wall, pancreas and kidney were the least injured, while the small bowel was the most commonly injured organ in penetrating injury 11(44%) patients. Overall, the spleen was the most commonly injured organ 36(26.5%) patients, followed by the small bowel 26(19.1%) patients and the liver 20(14.7%) patients. A total of 9 (6.6%) patients reported no visceral injury after laparotomy, and all of them had blunt abdominal injury (Fig. 2).

### Short-term outcomes in patients with abdominal injury

One hundred eighteen (86.8%) patients were alive, post-operative complications occurred in 57 (41.9%) patients, and 18 (13.2%) patients died.

The most common post-operative complication was surgical site infection (SSI) 20(28%) patients. A total of 15 (21%) patients had anaemia, and 41 (30.1%) patients had hypovolemic shock. Other complications were burst abdomen in 3 (5.3%) patients, intestinal obstruction due to adhesions in 3 (5.3%) patients, paralytic ileus 3 (5.3%) patients, comatose 1 (1.8%) patients and UTI 3 (5.3%) patients. Complications among 2 (3.5%) patients included intra-abdominal abscesses, convulsions, wound re-bleed and pneumonia (Table 2).

**Table 2:** Patient and clinical characteristics (*n*=136)

Characteristics	n	%
Age, median (IQR, in years)	31.5 (21.3–44.8)	
Age category (in years)		
≤ 20	31	22.8
21–40	67	49.3
> 40	38	27.9
Sex		
Male	115	84.6
Female	21	15.4
Education level of patient		
None	14	10.3
Primary	90	66.2
Secondary	24	17.6
Post-secondary	8	5.9
Patient’s insurance status		
Insured	13	9.6
Non	123	90.4
Mode of transport to hospital		
Ambulance	102	75.0
Private	8	5.9
Public	26	19.1
Mechanism of injury		
RTA	73	53.7
Fall from height	29	21.3
Gunshot	2	1.4
Stab	30	22.1
Other	2	1.5
Type of injury		
Blunt	99	72.8
Penetrating	37	27.2
Associated extra abdominal injury	89	65.4
Isolated abdominal injury	47	34.6
Haematocrit, mean (SD)	31.02(6.53)	
< 30	56	41.2
≥ 30	80	58.8
Systolic blood pressure		
< 90 mmHg	41	30.1
≥ 90 mmHg	95	69.9
Glasgow Coma Scale		
3–8 (severe)	5	3.7
9–12 (moderate)	11	8.1
13–15 (mild)	120	88.2
NISS		
< 16 (mild)	7	5.1
16–25 (moderate)	63	46.3
> 25 (severe)	66	48.5

### Factors associated with mortality

This study included 136 patients, 118 (86.8%) were alive and 18 (13.2%) died. On crude odds ratio, various clinical characteristics in abdominal injury patients and potentially confounding factors that affected the relationship between primary predictor variables and a dichotomous categorical outcome (dead or alive) were considered, with a 95% confidence interval and *P* < 0.05. Patients with associated extra-abdominal injuries, head injuries, pelvic injuries, a time interval > 6 h from injury to admission, being admitted for ≥7 days in the hospital (LOS), severe injury on the NISS, anaemia and systolic BP < 90 had higher odds of mortality, and these findings were statistically significant. Other factors, including age, sex, mechanism of injury, chest injury and extremity injury, were all associated with higher odds of mortality but were not statistically significant. After adjustment, severe injury on the NISS and a time interval > 6 h from injury to admission significantly predicted mortality (Table 3).

**Table 3:** Factors associated with mortality

Characteristics	T	Death n (%)	Alive n (%)	COR (95% CI)	*P*-value	AOR (95% CI)	*P*-value
Age in years							
≤ 20	31	2 (6.5)	29 (93.5)	Ref			
21–40	67	9 (13.4)	58 (86.6)	2.3 (0.5–11.1)	0.319		
> 40	38	7 (18.4)	31 (81.6)	3.3 (0.6–17.1)	0.159		
Sex							
Male	115	13 (11.3)	102 (88.7)	Ref			
Female	21	5 (23.8)	16 (76.2)	2.5 (0.8–7.8)	0.129		
Mechanism of injury							
Others	63	6 (9.5)	57 (90.5)	Ref			
RTA	73	12 (16.4)	61 (83.6)	1.9 (0.7–5.3)	0.241		
Associated Injuries							
No	47	2 (4.3)	45 (95.7)	Ref		Ref	
Yes	89	16 (18)	73 (82)	4.9 (1.1–22.5)	0.039*	0.4 (0.04–3.9)	0.415
Head injury							
No	94	7 (7.4)	87 (92.6)	Ref		Ref	
Yes	42	11 (26.2)	31 (73.8)	4.4 (1.6–12.4)	0.005*	3.1 (0.8–11.8)	0.104
Chest injury							
No	90	9 (10)	81 (90)	Ref			
Yes	46	9 (19.6)	37 (80.4)	2.2 (0.8–6.0)	0.126		
Pelvis injury							
No	124	14 (11.3)	110 (88.7)	Ref		Ref	
Yes	12	4 (33.3)	8 (66.7)	3.9 (1.0–14.7)	0.043*	2.6 (0.5–12.9)	0.235
Extremities							
No	99	11 (11.1)	88 (88.9)	Ref			
Yes	37	7 (18.9)	30 (81.1)	1.9 (0.7–5.3)	0.237		
Time to admission							
≤ 6	58	3 (5.2)	55 (94.8)	Ref		Ref	
> 6	78	15 (19.2)	63 (80.8)	4.4 (1.2–15.9)	0.025*	4.3 (1.0–18.9)	0.053*
Time to operation							
≤6	82	11 (13.4)	71 (86.6)	Ref			
>6	54	7 (13.0)	47 (87.0)	1.0 (0.3–2.7)	0.939		
NISS							
Mild	7	0	7 (100)				
Moderate	63	1 (1.6)	62 (98.4)	Ref			
Severe	66	17 (25.8)	49 (74.2)	21.7 (2.8–167.2)	0.003*	15.5 (1.5–160)	0.022*
Days in hospital (LOS)							
<7	121	13 (10.7)	108 (89.3)	Ref		Ref	
≥7	15	5 (33.3)	10 (66.7)	4.2 (1.2–14.0)	0.022*	1.6 (0.3–6.9)	0.507
Systolic blood pressure							
<90(Shock)	41	10 (24.4)	31 (75.6)	Ref		Ref	
≥90(Normal)	95	8 (8.4)	87 (91.6)	3.5 (1.3–9.7)	0.015*	0.6 (0.2–1.9)	0.360
SSI							
No	116	15 (12.9)	101 (87.1)	Ref			
Yes	20	3 (15.0)	17 (85.0)	1.2 (0.3–4.5)	0.801		
Anaemia							
No	115	11 (9.6)	104 (90.4)	Ref		Ref	
Yes	21	7 (33.3)	14 (66.7)	4.7 (1.6–14.2)	0.006*	2.8 (0.7–11.1)	0.128

*Statistically significant (*p* < 0.05), *COR* Crude odds ratio, *AOR* Adjusted odds ratio, *T* Total

## Discussion

Trauma is still causing a significant number of emergency visits globally. Abdominal trauma contributes significantly to the morbidity and mortality of trauma patients [17]. In this study, it accounted for 9.9% of all trauma patients seen during the study period, and this percentage was lower compared to figures reported by Chalya and coworkers who found a mortality rate of 17.5% among their 1678 cases [18]. In this study, we excluded patients on conservative management, patients who did not consent to participate, patients who died before their operations and patients who underwent operations in other health care units in this region. We excluded patients on conservative management because they are less likely to be referred to a consultant hospital and are more likely to be lost to follow-up. We understand that the exclusion of the remaining patients might exclude severely injured patients and lead to under-reporting of the true abdominal trauma cohort. There is no trauma registry in this region; hence, it is difficult to obtain trauma data from other health facilities and extrapolate the data for the region and the country. A true magnitude of the abdominal trauma cohort in our region requires the inclusion of data reported from other health care units, post-mortem reports and patients on conservative management.

The majority of patients were young people in the age group of 21–40 years, and there were more males than females. Individuals in this age group are more active in economic activities in society, and their involvement indicates a significant loss of production time in economic activities [18]. The majority of the patients (66.2%) had a primary school education and were subsistence farmers. This is a public health problem because it involves individuals in an economically active age group. Other studies with the same findings found a relationship to alcohol and drug misuse in this age group [19, 20]. The influence of drugs and alcohol in trauma victims was not studied in this study, so we do not know how it would have influenced the demography in this study.

In this study, the majority of patients (approximately three-quarters) had blunt abdominal injury; other studies reported similar findings [14]. Other studies reported the most common type of abdominal injury to be penetrating injury [21]. The high incidence of blunt abdominal injury in this study was attributed to the majority of patients being involved in RTAs, which occur because of increasing motorization, poor road infrastructure and urbanization in our locality. RTAs were the most common cause of abdominal injury. Similar findings were reported by other studies [22]. These findings are crucial to the establishment of preventive strategies to reduce RTAs and the subsequent incidence of trauma.

In this study, the majority of patients were brought in by ambulance services. This finding is contrary to those of other studies, in which very few patients were brought in by ambulance services in developing countries [14]. In this study, other patients were brought in by relatives, the police force, good Samaritans, and village and ward officials. The majority of patients brought in by ambulances had inadequate resuscitation, and those brought in by relatives, good Samaritans and village officials had no resuscitation. The pre-hospital care of trauma patients is important in trauma patient care, and it determines the outcomes after injury [23]. The absence of trauma rescue teams and properly trained personnel in peripheral hospitals to care for trauma patients have contributed to the poor outcomes of these patients. Other studies have shown that delayed injury arrival time highly contributes to the morbidity and mortality of trauma patients [10]. In our study, this observation was true; patients who were brought in more than 24 h after the injury came with complications. Trauma patients are referred together with other surgical and non-surgical emergencies. The absence of a coordinated trauma registry may cause patients to be taken to a health facility with no expertise in appropriate care. This may cause a delay in the initiation of definitive care and thus lead to complications for patients, similar to the findings of another study [23].

This evidence in our environment indicates a need for a coordinated trauma care system and properly trained personnel to take care of trauma patients, including patients with abdominal injuries. The presence of associated extra-abdominal injuries in addition to abdominal injuries causes more patients to have severe injury on the NISS and subsequently influences patients’ outcomes [24]. In this study, head injuries, chest injuries and extremity injuries were the most commonly injured parts, similar to findings in other studies [18]. This is attributed to the majority of the patients being involved in RTAs and falls from heights. The presence of associated extra-abdominal injuries and severe injuries were associated with high morbidity and mortality in this study. The spleen was the most commonly injured organ, followed by the liver and bowels, in blunt abdominal injury. Splenectomy was the most common means of treatment, followed by the repair of the liver and bowels. The gastrointestinal tract is commonly injured in penetrating abdominal injuries. These findings are similar to those of other studies [9, 25]. In studies reported from the developed world, splenectomy is not the most common method of treatment after splenic injury. Sixty to 90 % of patients with splenic injuries are managed conservatively [26]. This finding indicates the limitations in the performance of serial imaging and the availability of CT scan services in management protocols in such injuries, which lead to conservative management in LMICs. Trauma patients share these services with other emergency and elective patients in our facility.

The rate of negative laparotomy in this study was 6.6%, which was significantly low. The rate of negative laparotomy reported in other studies ranges from 7 to 40% [25]. Tertiary centres can receive selected patients from lower-level health facilities for operation. This study also has a selection bias, as it included only patients who had abdominal injury and were operated on, which can result in under-reporting of negative laparotomy.

Post-operative complications in our study occurred in 57 patients (41.91%) and was significantly higher than in other studies [9, 25]. This finding has an effect on the final outcomes of these patients. In our study, SSI was the most common post-operative complication and was attributed to bowel injury. The bowel contents spill into the peritoneal cavity, causing contamination of the peritoneal cavity (and subsequently the surgical wound) with gut bacteria. Similar findings have been documented in other studies [25].

The median (IQR) length of hospital stay in the general ward and ICU were 6 (2–9) and 2 (1–4) days, respectively. This finding was less than that reported by Chalya and coworkers who found an overall median length of hospital stay of 12 days among their 396 cases [14]. In our study, patients with severe NISS scores, associated extra-abdominal injury and post-operative complications had longer hospital stays. These hospital stays exacerbate the burden on resource-limited health care and reduce time for productivity among patients and their caretakers.

The mortality in this study was reported to be 18(13.2%) patients, which was higher than that found in other studies. In the literature, the mortality rate reported ranges from 7 to 40%. Other studies had similar mortality rates 17.1% [14] and 15% [25]. These studies had the same study design and study populations, which explain their similar results. Other studies had higher mortality 25.8% [27], this study had a higher rate of co-morbidities and hence higher mortality. Other studies [28] showed lower mortality rate of 4(4.7%) patients. Lower mortality rate was attributed to small study sample. We did not take into account the co morbidities in our patient’s population so it’s difficult to say how it would have affected the mortality in our study. In our study, the operation waiting time for trauma patients was longer. Trauma patients share the operating room with patients with other emergencies from surgery and orthopaedic and obstetric surgery, and they share imaging services with non-trauma emergency patients. Female gender was associated with a higher mortality rate. Five (27.8%) female patients died, which comprised approximately one-third of the overall mortality rate. These deaths were attributed to injury severity; all deceased female patients had severe injuries.

With respect to logistic regression in univariable analysis, patients with associated extra-abdominal injuries had 5 times higher odds of mortality, with a COR of 4.9 (1.1–22.5) (*P*-value 0.039). Among these patients, patients with head injury and pelvic injury had 4 times higher odds of mortality, with a COR of 4.4 (1.6–12.4) (*P*-value 0.005) and a COR of 3.9 (1.0–14.7) (*P*-value 0.043), respectively; this difference was statistically significant. Patients with severe injury on the NISS had higher odds of mortality, with a COR of 21.7 (2.8–167.2) (*P*-value< 0.003).

Patients with a time interval > 6 h from injury to admission and those with an LOS ≥7 days had 4 times higher odds of mortality, with a COR of 4.4 (1.2–15.9) (*P*-value 0.025) and a COR of 4.2 (1.2–14.0) (*P*-value 0.022), respectively. This difference was statistically significant. Mortality in patients with a time interval < 6 h from admission to the operation and LOS < 7 days was attributed to severe injury and severe bleeding. Patients who had RTAs and patients with chest injuries had 2 times higher odds of mortality, with a COR of 1.9 (0.7–5.3) (*P*-value 0.241) and a COR of 2.2 (0.8–6.0) (*P*-value 0.126), respectively, although this difference was not statistically significant.

Patients with an episode of systolic BP of < 90 had 4 times higher odds of mortality, with a *P*-value of 0.015 for the COR, which was significant. Patients with anaemia had 5 times higher odds of mortality, with a *P*-value of 0.006 for the COR. In multivariable analysis, after adjustments were made for other factors, severe injury on the NISS and a time interval > 6 h from injury to admission significantly predicted mortality, with aORs of 15.5 (95% CI: 1.5–160, *P*-value< 0.022) and 4.3 (95% CI: 1.0–18.9, *P*-value< 0.053), respectively. The *P*-value of interval time > 6 h from injury to admission was marginally significant.

Knowledge of the factors associated with mortality in our setting is crucial in reducing the mortality associated with abdominal injury. Of all deaths, one-third of patients (33.3%) died within 48 h; these deaths were attributed to inadequate resuscitation and overwhelming injuries. Half of them (9 patients; 50.0%) died during the first 7 days, and these deaths were attributed to bleeding and infections. The other 3 patients (16.7%) died days to weeks after the injury, and these deaths were attributed to infections and multi-organ failure. The identification of factors associated with morbidity and mortality in our region is important in reducing morbidity and mortality associated with abdominal injury.

The exclusion of patients not consented to participate, patients on conservative management, died prior to operation, patients who were operated in other health facilities and inclusion of patients only proceeded to surgery were limitations and may cause selective bias in this study. Small number of study participants may make differences detected among study groups to be interpreted with caution.

## Conclusion

In this study, blunt abdominal injury was the most common type of injury. Splenic injury was the most common pattern, while RTAs were the most common cause of injury. Younger people were commonly affected, and the most common complications were SSI, hypovolemic shock and anaemia. Mortality was highly associated with associated extra-abdominal injuries, delays in coming to the hospital, and severe injuries. The establishment of a coordinated trauma registry and system of care and evacuation teams is highly recommended in our settings and in LMICs to curb the prevailing morbidity and mortality. Furthermore, larger multicentre studies are needed to evaluate the trauma care capability of health care facilities in our region and the impact of trauma to our population. We recommend bedside imaging for trauma patients in LMICs to avoid longer waiting times for operations.

## Data Availability

Data will be available upon contacting corresponding author on reasonable request.

## Abbreviations

AOR: Adjusted odds ratio
ATLS: Advanced trauma life support
BP: Blood pressure
COR: Crude odds ratio
ICU: Intensive care unit
IQR: Inter-quartile range
KCMC: Kilimanjaro Christian Medical Centre
LMICs: Low- and middle-income countries.
LOS: Length of hospital stay
NISS: New Injury Severity Score
RTA: Road traffic accident
